# Psychodynamic accessibility: a testable framework for supported agency in social psychiatry and psychiatric rehabilitation

**DOI:** 10.3389/fpsyt.2026.1865419

**Published:** 2026-06-12

**Authors:** Eik Niederlohmann

**Affiliations:** Department of Psychosomatic Medicine and Psychotherapy, Kliniken Erlabrunn, Breitenbrunn, Germany

**Keywords:** capability approach, epistemic trust, psychiatric rehabilitation, psychodynamic accessibility, recovery, social psychiatry, supported decision-making, supported employment

## Abstract

Recovery-oriented social psychiatry and psychiatric rehabilitation increasingly emphasize rights, participation, community-based care, supported decision-making, supported employment, and prevention-oriented early intervention. Yet formal access to services, information, rehabilitation, legal-administrative procedures, or participation formats does not by itself ensure that people can use these resources when shame, mistrust, affective overload, fatigue, cognitive narrowing, or structural vulnerability are already present. This Hypothesis and Theory article introduces psychodynamic accessibility as a provisional middle-range mechanism for the formal-access/effective-agency gap. Psychodynamic accessibility refers to the degree to which services, rehabilitation pathways, workplaces, administrative procedures, and prevention-oriented access pathways remain usable when affect tolerance, self-worth, mentalizing, epistemic trust, defensive functioning, or personality functioning are strained by the setting itself. The article does not propose a general theory of socioeconomic inequality or service disengagement. Its narrower aim is to define psychodynamic conversion factors that may influence whether formal resources are converted into effective agency in mental health care, psychiatric rehabilitation, supported employment, supported decision-making, and secondary or tertiary prevention access pathways. Building on the capability approach, rights-based community mental health, recovery theory, social-determinants research, trauma-informed and procedural-justice frameworks, early-intervention service models from Australia and Canada, epistemic-trust research, and psychodynamic accounts of functioning under stress, the manuscript derives six candidate conversion factors: shame and punitive self-evaluation, defensive functioning under threat, affect tolerance and symbolization thresholds, personality functioning and structural integration, epistemic mistrust or credulity, and dependence on external organizers. The central hypothesis is that developmental adversity, precarious work, stigma, and opaque institutional procedures can become self-reinforcing when these factors reduce help-seeking, service engagement, rights-claiming, shared decision-making, and self-advocacy. Psychodynamic accessibility reframes inclusion as the design of low-arousal, dignity-preserving, relationally continuous, epistemically trustworthy, procedurally comprehensible, temporally flexible, and rights-compatible environments. The article specifies selection criteria, competing explanations, boundary conditions, failure criteria, and a staged validation roadmap from qualitative mapping to measurement development, service-redesign pilots, implementation studies, and secondary or tertiary prevention outcomes. The model does not diagnose social groups as deficient. It identifies person-in-context barriers and institutional design levers for supported effective agency.

## 1 Introduction: the formal-access/effective-agency gap

For social psychiatry, the critical test of access begins not when a referral, right, or rehabilitation program exists, but when a distressed person must use it. A person may formally have a service referral, legal capacity, a complaint procedure, an employment right, a treatment recommendation, or an opportunity to participate in rehabilitation. Yet the route into these resources may require stable self-worth, verbal abstraction, future orientation, trust in unfamiliar authorities, shame tolerance, and self-advocacy under arousal. In that situation, access is present in the administrative sense but unavailable in the functional and experiential sense (1–11, 50, 51).

This article reads access from the point of use backward: not from the existence of a right, but from the transition at which a person must convert that right into action. The guiding question is therefore not only whether a service exists, but whether the service remains usable when the person is ashamed, mistrustful, fatigued, defensive, cognitively narrowed, or already socially exposed. This question is central for psychiatric rehabilitation because rehabilitation aims not merely to reduce symptoms, but to support participation, identity, hope, agency, and community life despite continuing vulnerability (3, 8–11).

The proposed term for this problem is psychodynamic accessibility. Psychodynamic accessibility refers to the degree to which institutions, services, workplaces, legal-administrative procedures, and participation formats are designed so that people can exercise agency even when affect tolerance, self-worth, mentalizing, epistemic trust, symbolization, defensive functioning, or personality functioning are temporarily or chronically strained. The term does not identify a new diagnostic group. It identifies a class of affective, relational, epistemic, temporal, procedural, and self-evaluative barriers that may prevent formal access from becoming effective agency.

The construct is anchored in the capability distinction between resources and real opportunities for doing and being (1, 2). Hopper translated this distinction into social recovery in schizophrenia by asking how recovery depends on real capability rather than on nominal access alone (3). Psychodynamic accessibility adds a more specific mechanism layer: some conversion barriers are not only material, cognitive, or legal, but also affective, defensive, relational, epistemic, and self-evaluative. These barriers do not replace poverty, discrimination, racism, gendered violence, migration precarity, labor-market insecurity, housing shortage, or inadequate service capacity as explanations. They describe one way in which such conditions may become embodied, enacted, and institutionally amplified.

The practical implication is direct. A rights-based and recovery-oriented system is not fully accessible if it requires psychologically ideal users before people are able to participate. Services can be physically reachable, legally available, digitally searchable, and verbally explained while still being inaccessible to people who cannot tolerate humiliation, maintain trust, organize time, or advocate for themselves under pressure. Social psychiatry therefore needs a vocabulary for the transition between formal access and supported effective agency. This article proposes psychodynamic accessibility as that vocabulary.

## 2 Purpose, scope, non-goals, and reading path

The purpose of this article is to introduce and delimit psychodynamic accessibility as a candidate mechanism layer between formal access and effective agency in recovery-oriented social psychiatry and psychiatric rehabilitation. The article has four aims: to define the construct, derive its components, formulate testable hypotheses, and outline a staged validation agenda. The argument is theoretical, not a report of original empirical data.

The intended scope is deliberately narrower than the domains touched by the surrounding literature. The primary application fields are mental health services, psychiatric rehabilitation, supported employment, supported decision-making, and legal-administrative interfaces. Prevention-oriented implications are included where they concern secondary or tertiary prevention: early engagement, early psychosis or youth-service pathways, reduction of dropout, re-entry after disengagement, and prevention of service-induced deterioration. Broader population-communication, educational, and cultural applications remain outside the evidentiary core of the framework and would require separate validation.

The article does not propose a general theory of socioeconomic inequality, institutional distrust, or labor-market behavior. It does not classify precarious workers, service users, or persons with psychosocial disabilities as deficient. It does not replace social-determinants research, disability rights, or structural analyses of racism, sexism, ableism, migration precarity, housing markets, labor law, or service capacity. Psychodynamic accessibility is a complementary person-in-context mechanism, not a total explanation.

The manuscript is built as a learning path. Section 3 explains the derivational logic and selection criteria. Section 4 situates the construct within rights-based, recovery-oriented, capability, prevention, and access literatures. Sections 5 and 6 define the design dimensions and conversion factors. Section 7 formulates hypotheses, competing explanations, and boundary conditions. Sections 8 and 9 translate the framework into application domains and staged validation. Sections 10–13 specify ethical safeguards, advantages, limitations, and the final discussion. This architecture is intended to make the theoretical status of each claim explicit and to prevent conceptual accumulation from being mistaken for validation.

## 3 Derivation of the model

The model is derived from one problem: formal access does not automatically become effective agency. The capability approach supplies the first distinction: resources, rights, and opportunities require conversion conditions before they become real freedoms (1–3). Rights-based community mental health supplies the second layer: autonomy, accessibility, legal capacity, community inclusion, and freedom from discrimination require service environments that can be used in practice (4–7). Recovery theory supplies the third layer: meaningful agency is connected to hope, identity, connection, purpose, and empowerment, not only to symptom reduction (8–11). Social-determinants and precarious-employment research supply the fourth layer: adversity and insecurity shape the conditions under which people can engage with services, work, and institutions (19–26). Psychodynamic and mentalization-informed theory supplies the mechanism layer: affect tolerance, self-worth, defense, symbolization, personality functioning, and epistemic trust influence how people learn, relate, and act under threat (27–33, 36–42).

**Figure 1:**
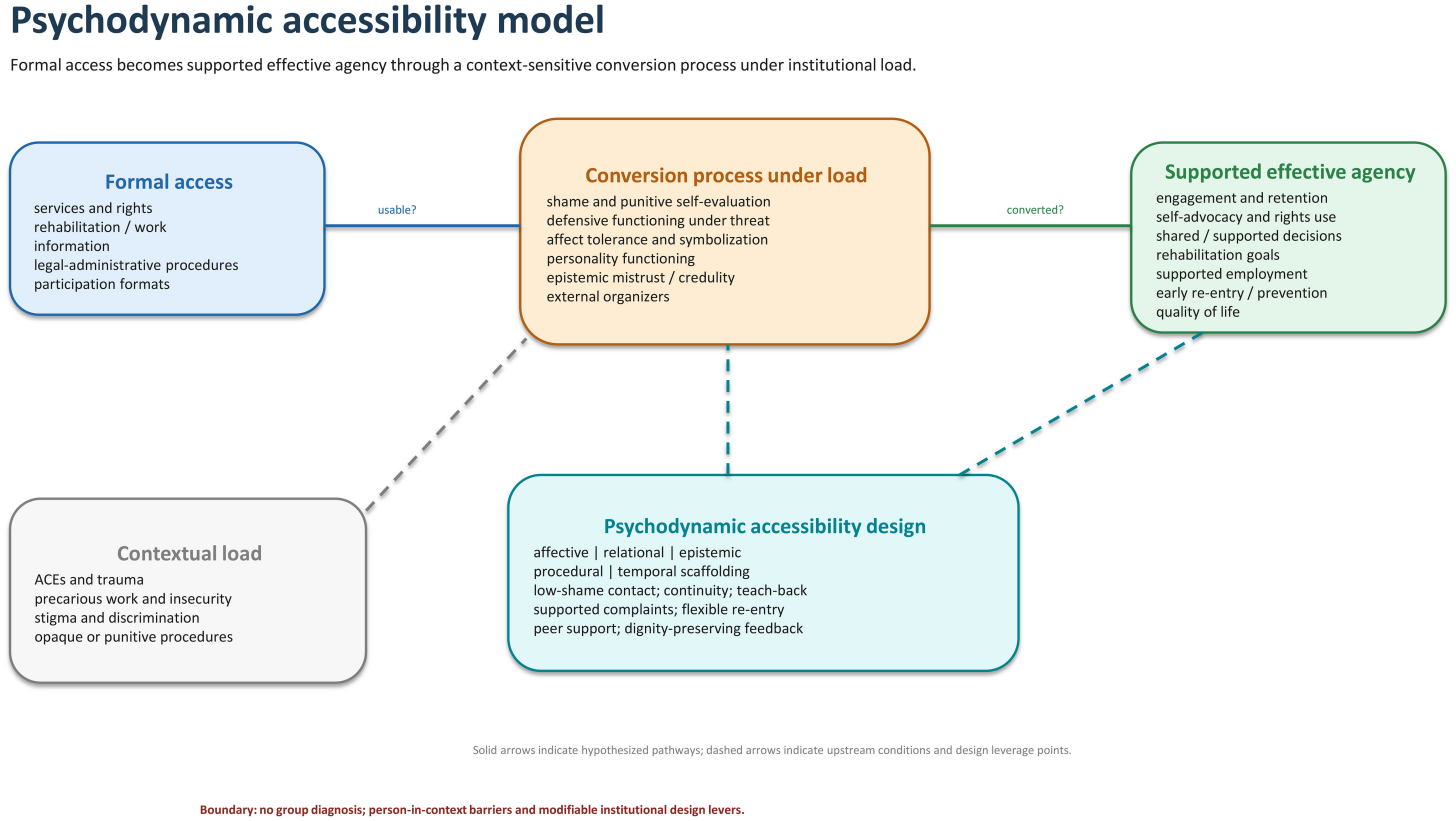
Psychodynamic accessibility as a provisional middle-range mechanism. The model integrates the distinction between resources and real capabilities from the capability approach, rights-based community mental health and recovery-oriented care, social-determinants and administrative-burden research, psychodynamic/personality-functioning accounts of functioning under stress, epistemic-trust research, supported decision-making, procedural justice, supported employment, international early-intervention/youth-hub models, implementation science, and trauma-informed care (1–11, 19–35, 43–56). Solid arrows indicate hypothesized pathways from formal access through conversion processes under load to effective agency; dashed arrows indicate design leverage points. The model does not classify social groups as deficient; it identifies person-in-context barriers and modifiable institutional conditions.

The proposed factors were not selected as a broad list of interesting clinical concepts. They were selected because they meet six criteria: capability relevance, state dependence under institutional load, clinical or service-level observability, modifiability by institutional design, non-stigmatizing person-in-context interpretation, and measurement plausibility. **Table 1** summarizes these criteria. **Figure 1** summarizes the resulting mechanism: formal access becomes supported agency only through a conversion process under load, and institutional design can either intensify or scaffold that process.

**Table 1:** Selection criteria for psychodynamic conversion factors and design dimensions.

Criterion	Operational meaning	Examples of evidence or indicators
Capability relevance	The factor must plausibly influence whether a resource, right, service, or opportunity becomes a real capability.	Dropout, rights-claiming, self-advocacy, supported employment, shared decision-making.
State dependence under load	The factor must vary with stress, institutional pressure, humiliation, fatigue, or relational threat.	Within-person change across intake, crisis, discharge, complaint, job interview, or administrative deadlines.
Clinical or service observability	The factor must be visible in clinical process, service engagement, communication, or functional behavior.	Missed appointments, ruptures, avoidance, concrete thinking, submission, aggression, somatic discharge.
Modifiability by design	The factor must be at least partly influenced by environmental redesign, not only by individual treatment.	Low-shame letters, relational continuity, teach-back, peer support, flexible scheduling, procedural justice.
Non-stigmatizing interpretation	The factor must be formulated as person-in-context functioning rather than as a group deficit.	Context-sensitive descriptions, anti-stigma safeguards, lived-experience review.
Measurement plausibility	The factor must have candidate instruments or observable indicators suitable for staged validation.	ETMCQ, OPD-3, shame/self-criticism scales, engagement metrics, implementation outcomes.

This derivation also clarifies what kind of model is being proposed. Psychodynamic accessibility is not a diagnostic taxonomy, a service-quality checklist, or a causal model already ready for broad institutional rollout. It is a provisional middle-range framework. Its value depends on whether future work can show that the proposed conversion factors add explanatory or intervention value beyond objective service availability, socioeconomic resources, diagnosis, symptom severity, cognitive burden, and standard procedural complexity.

The model therefore has a developmental status. At this stage, it should be used to generate hypotheses, organize qualitative mapping, design candidate measures, and structure low-risk service redesigns. Only after empirical testing should it be used as a basis for implementation decisions. This staged logic is essential because a theory of accessibility can itself become harmful if institutions use it to label people as fragile rather than redesigning inaccessible procedures.

**Table 1** and **Figure 1** summarize the selection logic and the primary pathway, respectively.

## 4 Current discourse: rights, recovery, capability, prevention, and hidden barriers

Rights-based mental health frameworks emphasize dignity, freedom from discrimination, equal recognition before the law, supported decision-making, accessibility, and community-based alternatives to coercive or institution-centered care (4–7). The Convention on the Rights of Persons with Disabilities places accessibility and the supported exercise of legal capacity at the center of disability rights (7). The WHO QualityRights program and related guidance similarly emphasize rights, person-centeredness, community inclusion, and the reduction of coercive practices (4–6). Psychodynamic accessibility is intended to complement this agenda. It asks how rights and services remain usable when people are overwhelmed, ashamed, distrustful, or unable to self-advocate in the idealized way institutions often expect.

Recent contributions in the target section illustrate the same service-design concern. A QualityRights recovery program study in South Korea emphasized participation, engagement, communication, decision-making, mutual understanding, and the redefinition of recovery aims (12). A qualitative case study of Medication-Free services described clashes between flexible recovery intentions and inflexible ward structures, power imbalances, and insufficient relational work (13). A European perspective on psychosocial disability highlighted housing, employment, healthcare access, social inclusion, organizational culture, and stigma (14). A discourse analysis of young people with severe mental health problems and complex needs described how traditional services can place people into boxes and how flexible assertive community treatment can offer a less rejecting alternative (15). These studies do not validate the present model, but they show that organizational culture, power, continuity, and relational usability are central rather than peripheral in rehabilitation.

International prevention and early-intervention models add a second important context. Australian early psychosis services and headspace show how secondary prevention can become a service architecture rather than a single intervention: youth-friendly entry, rapid response, work-and-study support, online and community access, and a non-stigmatizing service identity are combined to reduce delay and disengagement (45–47). Canadian and international integrated youth service hubs similarly emphasize low-threshold, community-based, multi-component access, while Kids Help Phone demonstrates the preventive value of anonymous, remote, and post-based support as a lower-shame entry pathway (48, 49). Psychodynamic accessibility does not replace these models. It specifies an often implicit layer within them: the affective, relational, epistemic, and temporal design conditions that make early help usable before avoidance, distrust, or shame-driven dropout become entrenched.

Access research also supports the need to study the interface between systems and populations rather than service supply alone. Patient-centered access has been conceptualized as a fit between the abilities of people and the features of services, including approachability, acceptability, availability, affordability, and appropriateness (50). Administrative-burden research makes a related point for citizen-state interactions: learning costs, psychological costs, and compliance costs can block the use of public benefits even when entitlements formally exist (51). Sludge research similarly describes excessive frictions that stop people from completing necessary action (52). Psychodynamic accessibility adds that some of these burdens are not merely informational or procedural. They may also activate shame, mistrust, defensive rigidity, or self-attack.

## 5 Definition and design dimensions

A setting is psychodynamically accessible when it can be used by persons whose affect tolerance, self-worth, mentalizing, epistemic trust, defensive organization, or personality functioning are strained by the setting itself. A service can be physically accessible, legally available, digitally reachable, and verbally explained, yet psychodynamically inaccessible if its procedures require high arousal tolerance, abstract language, trust in unknown authorities, stable self-esteem, and persistent self-advocacy after humiliation or threat.

Five design dimensions follow from this definition. Affective accessibility means reducing unnecessary shame exposure, threat, and arousal. The question is whether distress can become speakable before it becomes action, withdrawal, aggression, somatization, or dropout. Affective accessibility includes pacing, non-shaming language, predictable procedures, de-escalation, and time to recover from high-arousal encounters. The rationale is consistent with stress research showing that acute stress can impair prefrontal control and with organizational threat-rigidity theory, which describes simplification, narrowing, and routine-bound behavior under threat (27, 28).

Relational accessibility means continuity, acknowledgment, and the availability of a human relationship through which the person can orient. Recovery-oriented and QualityRights approaches repeatedly emphasize participation, communication, mutual understanding, personal meaning, shared decision-making, and human connection (4–13). A relationally inaccessible service may offer appointments but no one who remembers the person, notices shame, or repairs rupture. Relational accessibility is not sentimental. It is a structural condition for engagement.

Epistemic accessibility means that information can be trusted, questioned, repeated, and integrated. Mentalization theory describes how the capacity to learn from others depends on epistemic trust: an openness to socially communicated knowledge as personally relevant and reliable (29, 30). The Epistemic Trust, Mistrust and Credulity Questionnaire operationalizes trust, mistrust, and credulity, including a validated German version (31, 32). A balanced state of epistemic trust is neither naive trust nor cynical suspicion; it is the ability to remain learnable while evaluating sources. Epistemic accessibility therefore does not ask people to trust institutions blindly. It designs communication that recognizes legitimate mistrust, separates uncertainty from manipulation, and keeps people able to ask questions without humiliation.

Procedural accessibility means that steps, timeframes, consequences, rights, appeals, and feedback are comprehensible and proportionate. Procedural justice emphasizes voice, neutrality, respect, and trustworthiness as conditions for engagement and perceived legitimacy, including in forensic mental health contexts (34). Supported decision-making reframes legal capacity as something exercised with support rather than replaced by paternalistic control (35). Psychodynamic accessibility extends this logic: procedures should be designed for people who may become concrete, submissive, defensive, dissociated, or overwhelmed under institutional pressure.

Temporal accessibility means that institutions do not assume unlimited time, stable attention, regular sleep, predictable availability, or a linear path from information to action. Shift work, night work, commuting, caregiving, chronic pain, insecure housing, substance use, appointment fatigue, and depression all reduce the capacity to attend appointments, fill forms, participate in therapy, or deliberate. Temporal accessibility includes flexible scheduling, reminders, repeated contact, and low-penalty re-entry after missed appointments or dropout. **Table 2** provides examples of hidden psychological entry requirements and corresponding accessibility responses.

**Table 2:** Hidden psychological entry requirements and psychodynamic accessibility responses.

Hidden entry requirement	Where it appears	Psychodynamic accessibility response
Stable self-worth and shame tolerance	Applications, interviews, wage negotiation, complaints, therapy intake	Normalize support; use low-shame language; offer rehearsed self-advocacy; protect dignity after errors
High verbal abstraction under arousal	Courts, welfare procedures, medical consent, rehabilitation planning	Concrete steps; repetition; visual summaries; teach-back; human contact; gradual pacing
Trust in unfamiliar authorities	Service and prevention messages, psychiatry, benefit offices, courts	Transparent uncertainty; peer mediation; continuity; procedural justice; opportunities to question
Future orientation and planning	Appointments, deadlines, treatment plans, education, rehabilitation participation	Reminders; flexible scheduling; low-penalty re-entry; scaffolded routines; outreach
Self-advocacy under threat	Legal claims, workplace rights, shared decision-making, administrative complaints	Supported decision-making; coached assertion; immediate comprehensible feedback; non-retaliatory complaint pathways
Internal organization without external structure	Sick leave, unemployment, basic income, service transitions	Replacement scaffolds: peer support, supported employment, predictable routines, community anchors

## 6 Psychodynamic conversion factors under institutional load

Psychodynamic conversion factors are intrapsychic and relational processes that influence whether formal goods - services, money, information, rights, work opportunities, treatment plans, and participation formats - become effective agency. They are called conversion factors by analogy with the capability approach (1–3). The factors are not social labels. They are state- and context-sensitive mechanisms that may become visible when institutional demands exceed the person’s available regulatory, relational, or epistemic resources.

The first factor is shame and punitive self-evaluation. Harsh self-critical internal models can block self-advocacy, help-seeking, wage negotiation, complaint behavior, and application behavior. A person who experiences self-presentation as fraud, ambition as arrogance, and help-seeking as shameful will have lower effective access to education, employment, therapy, and rights even when formal opportunities exist. In contemporary, less stigmatizing language, these processes can be described as punitive self-evaluative systems rather than as fixed character deficits.

The second factor is defensive functioning under threat. Defensive processes influence how conflict, dependency, aggression, shame, and fear are processed. Vaillant’s work on defense mechanisms offers a long-standing developmental and clinical framework, while Snarey and Vaillant’s longitudinal study connected ego defenses with upward social mobility alongside IQ, maternal education and occupation, and boyhood ego strength (39, 40). This literature does not imply that people are responsible for poverty. It suggests that defensive capacities may shape how social opportunities are approached, avoided, or made unusable under threat.

The third factor is affect tolerance and symbolization threshold. Bion and Winnicott described early containment, symbolization, thinking, and play as developmental conditions for reflective functioning (41, 42). In service terms, thresholds are the points at which affective arousal narrows or collapses symbolic functioning. Below threshold, the person can think, feel, relate, and choose. Beyond threshold, the person may become concrete, dissociative, rigid, blank, ashamed, submissive, aggressive, or somatic. Prior clinician-facing process frameworks by the author modeled related state-dependent domains for psychotherapy and functional documentation: one article described reduced expressivity and withdrawal as context-sensitive capacity signals rather than fixed deficits, while two further frameworks linked defensive organization, affect tolerance, progression, and shame to safety-gated clinical action (16–18). The present article extends that process logic from the therapy room to institutional access.

The fourth factor is personality functioning and structural integration. The third version of Operationalized Psychodynamic Diagnosis (OPD-3) provides a clinically established framework for assessing personality functioning, conflicts, and structure (36). OPD-related work supports the distinction between psychodynamic conflicts and personality functioning while showing that they are associated in clinically meaningful ways (37, 38). In the present model, reduced structural integration is not a stigmatizing social label. It describes a person- and state-specific vulnerability of functions such as affect differentiation, self-regulation, mentalization, and relational continuity. These vulnerabilities may become visible only under load.

The fifth factor is epistemic mistrust or credulity. Generalized mistrust can turn correction into humiliation and information into suspected manipulation. Credulity can make emotionally coherent but false narratives more attractive than uncertain, complex institutional communication. Empirical work on epistemic trust, mistrust, and credulity makes this construct measurable and links it to psychopathology and social learning (31–33). The practical question is whether services can remain credible without demanding submission, and whether they can acknowledge uncertainty without losing reliability.

The sixth factor is dependence on external organizers. Work routines, institutional roles, religious groups, military structures, family obligations, public status, and professional identity may provide scaffolding for people whose internal organization is fragile. The problem arises when systems mistake dependence on external structure for free choice. A person may continue exhausting work because the work is the only available organizer of time, identity, and self-worth. Conversely, sudden removal of structure may produce collapse rather than freedom. Psychodynamic accessibility therefore favors scaffolded autonomy: external supports should become more dignifying, flexible, and self-chosen rather than abruptly removed or coercively intensified. **Table 3** summarizes the conversion factors, mechanisms, and measurement options.

**Table 3:** Psychodynamic conversion factors, candidate mechanisms, and measurement options.

Conversion factor	Candidate mechanism	Possible measures or indicators
Shame and punitive self-evaluation	Self-advocacy feels fraudulent, arrogant, disloyal, or dangerous.	Self-criticism and shame scales; observed avoidance of help-seeking, complaint, application, or negotiation tasks.
Defensive functioning under threat	Conflict is managed by splitting, denial, projection, dissociation, somatization, or intellectualization rather than reflective action.	Defense measures; clinician ratings; rupture markers; high-stakes task observation.
Affect tolerance and symbolization threshold	Institutional load triggers arousal, concreteness, dropout, aggression, somatic discharge, or collapse.	Anxiety-channel observation; physiological arousal; missed appointments; narrative concreteness; recovery after rupture.
Personality functioning/structural integration	Self-regulation, mentalization, and relational continuity fail under load.	OPD-3 structure axis; LPFS/STIPO-type measures; ICF functioning domains; relational-continuity indicators.
Epistemic mistrust or credulity	Correction feels humiliating or manipulative; emotionally coherent false narratives become attractive.	ETMCQ; institutional trust; misinformation discrimination tasks; response to transparent uncertainty.
Dependence on external organizers	Work or rigid roles stabilize identity and time while constraining autonomy.	Occupational history; response to sick leave, unemployment, discharge, service transitions, or supported-employment scaffolds.

## 7 Hypotheses, competing explanations, and boundary conditions

The model can be summarized in five testable hypotheses. The hypotheses treat psychodynamic accessibility not as a property of persons, but as a testable person-environment fit. They predict that changing institutional design - not merely treating individuals - can change engagement, rights use, self-advocacy, rehabilitation participation, and re-entry after dropout.

Hypothesis 1 is the psychodynamic conversion hypothesis. Developmental adversity, socioeconomic precarity, stigma, and institutional opacity are associated with reduced effective agency in services, employment, law, and community participation. This association is partly mediated or moderated by shame, harsh self-criticism, defensive functioning under threat, low affect tolerance, personality-functioning vulnerabilities, epistemic mistrust or credulity, and dependence on external organizers.

Hypothesis 2 is the hidden-entry-requirement hypothesis. Institutions that require high verbal abstraction, stable self-worth, future planning, trust, and self-advocacy under arousal will be less usable for persons with state-dependent structural vulnerability, even when formal access is equal. Psychodynamically accessible procedures should improve engagement, retention, rights-claiming, and satisfaction particularly among persons with high shame, mistrust, or affect dysregulation.

Hypothesis 3 is the self-advocacy blockade hypothesis. Harsh self-critical internal models and shame proneness predict reduced wage negotiation, complaint behavior, help-seeking, application behavior, and administrative contacting above and beyond income, education, symptom severity, and objective opportunity. This hypothesis tests whether punitive self-evaluation functions as a mobility brake.

Hypothesis 4 is the external-organizer hypothesis. For some structurally vulnerable persons, work, institutional roles, or rigid routines function as stabilizing external organizers. Interventions that reduce coercive work demands but do not provide replacement scaffolding may improve objective freedom while worsening subjective fragmentation, dropout, depression, or substance use in a subgroup. Conversely, supported employment, relational continuity, peer structures, and predictable routines may convert formal freedom into usable autonomy.

Hypothesis 5 is the epistemic accessibility hypothesis. Under perceived threat, service, prevention, and administrative communication is less effective when it relies only on factual correction and more effective when it also addresses shame, mistrust, humiliation, uncertainty, and source legitimacy. Epistemic trust, mistrust, and credulity should moderate responses to service messages, legal procedures, welfare institutions, rehabilitation planning, and prevention-oriented access pathways.

The model is falsifiable only if competing explanations and exclusion criteria are specified. **Table 4** lists operational tests, competing explanations, critical controls, and failure criteria. The model would be weakened if the proposed conversion factors add no predictive, mediating, moderating, or implementation value beyond objective access, socioeconomic resources, symptom severity, diagnosis, cognitive burden, and standard procedural complexity. The model would also be weakened if low-shame, relational, epistemically transparent, temporally flexible, and procedurally supported redesign do not improve outcomes in the groups predicted to benefit most.

**Table 4:** Hypothesis audit: operationalization, competing explanations, controls, and failure criteria.

Hypothesis	Operational test	Competing explanation	Critical failure criterion
H1 Psychodynamic conversion	Model ACEs, precarity, stigma, and institutional opacity as predictors; test shame, self-criticism, affect tolerance, defensive functioning, personality functioning, and epistemic mistrust as mediators or moderators of agency outcomes.	Dropout is fully explained by resources, diagnosis, symptom severity, cognition, service capacity, or waiting time.	No additional predictive, mediating, or moderating value of conversion factors after social, clinical, cognitive, and service controls.
H2 Hidden entry requirements	Compare standard vs psychodynamically accessible procedures for intake, complaints, rehabilitation planning, or supported decision-making.	Barriers are purely informational or procedural, not affective or relational.	Low-shame, concrete, relational procedures do not improve engagement or rights-claiming among high-shame or high-mistrust groups.
H3 Self-advocacy blockade	Test whether self-criticism and shame predict reduced applications, wage negotiation, complaint behavior, legal help-seeking, or administrative contacting.	Self-advocacy is explained only by income, education, legal knowledge, opportunity, or labor-market structure.	Self-criticism and shame show no independent or interaction effect after objective opportunity and socioeconomic controls.
H4 External organizer	Study transitions around sick leave, unemployment, supported employment, discharge, or change in work demands; identify subgroups for whom structure loss increases distress or dropout.	Structure loss affects outcomes only through income loss, symptom burden, or substance use.	Replacement scaffolding does not moderate outcomes after structure loss.
H5 Epistemic accessibility	Experimentally compare factual correction alone with service or prevention messages that acknowledge uncertainty, mistrust, shame, and source legitimacy.	Message effects are explained only by education, media use, procedural complexity, or prior institutional trust.	Epistemic-trust variables do not moderate message effects, or enhanced messages perform no better than facts alone.

The model does not explain cases in which formal access is genuinely absent, service capacity is insufficient, legal rights are denied, coercion or violence is primary, or severe neurocognitive impairment or acute psychosis makes supported agency impossible without more intensive intervention. It also does not explain all dimensions of poverty, social exclusion, precarious work, or service disengagement. Those phenomena require structural, legal, epidemiological, and sociological explanations. Psychodynamic accessibility addresses only the conversion layer at which existing opportunities become usable or unusable under load.

## 8 Application domains and prevention-oriented implementation

Mental health services and psychiatric rehabilitation are the primary domains. Services can examine whether intake, waiting-list, appointment, medication, psychotherapy, rehabilitation, discharge, and complaint procedures assume capacities that many users lack under stress. A person may be labeled non-adherent when the actual barrier is shame, fragmented memory, distrust, arousal-driven narrowing, or fear of being boxed into a stigmatizing category. Psychodynamic accessibility would redesign these points of contact around low-shame re-entry, concrete next steps, named contact persons, relational continuity, teach-back, and dignified repair after missed appointments.

Supported employment and occupational rehabilitation are the second domain. Precarious workers and people with psychosocial disabilities may need more than job training or motivational counseling. They may need support with shame, self-presentation, wage negotiation, complaint procedures, and recognition of exploitative patterns. Individual Placement and Support is the strongest international best-practice model for converting formal labor-market participation into actual employment outcomes through rapid competitive placement, integration with mental health care, and follow-along support (44). Psychodynamic accessibility adds specific modules for low-shame applications, coached self-advocacy, affect pacing during interviews, and the careful replacement of coercive external organizers with chosen structure.

Legal, administrative, and supported decision-making interfaces form the third domain. Legal and administrative systems often assume that a person can understand delayed consequences, tolerate complex language, remember deadlines, narrate facts coherently, and assert rights while anxious. Psychodynamic accessibility would favor timely, concrete, proportionate, non-humiliating, and procedurally just feedback embedded in support (34, 35, 51, 52). This does not mean abandoning autonomy or due process. It means recognizing that supported legal capacity requires psychological and relational scaffolding.

Prevention-oriented implementation is the fourth domain, but it must be carefully delimited. The present model is most relevant to secondary and tertiary prevention: early engagement before deterioration, reduction of dropout, prevention of service-induced shame or distrust, relapse-sensitive re-entry, and stabilization of participation after crisis. Australian early intervention and headspace models demonstrate that early access can be organized as a youth-friendly, community-based, and low-stigma system rather than as a narrow clinic pathway (45–47). Integrated youth service hubs and anonymous support formats such as Kids Help Phone show how multichannel access can reduce stigma, distance, and threshold barriers (48, 49). Psychodynamic accessibility translates these lessons into an explicit design question: does the pathway reduce shame, preserve epistemic trust, offer temporal flexibility, and support agency before avoidance becomes chronic?

Other fields - including broader population communication, education, and cultural participation - are not developed as application domains in this article. They may become later extension fields only after the core model has been validated in mental health services, rehabilitation, supported employment, supported decision-making, and prevention-oriented access pathways.

## 9 Operationalization and staged validation roadmap

The model is testable with mixed methods, longitudinal designs, implementation studies, and service-redesign trials. Candidate predictors include adverse childhood experiences, socioeconomic precarity, subjective social status, precarious employment indicators, shame, self-criticism, defensive functioning, personality functioning, affect tolerance, epistemic trust, perceived procedural justice, administrative burden, and institutional trust. Candidate outcomes include service engagement, dropout, missed appointments, complaint behavior, legal help-seeking, job-search self-efficacy, wage negotiation, supported employment outcomes, shared decision-making, quality of life, prevention-message comprehension, and community participation.

Possible instruments include the ETMCQ for epistemic trust, mistrust, and credulity (31, 32), OPD-based measures for personality functioning and conflict (36–38), ACE measures (23–26), precarious employment scales and multidimensional indices (20–22), ICF-based functioning assessment, shame and self-criticism scales, procedural-justice indicators, administrative-burden measures, and standardized service-engagement indicators. In clinical-process research, CSA and PAD-S offer structured ways to record observable states - defense, affect tolerance, progression, and shame - and link them to functional consequences (17, 18).

Qualitative work should come first. Interviews and participatory mapping with service users, people with psychosocial disabilities, precarious workers, early-intervention users, peer workers, case managers, supported-employment specialists, and administrative staff can identify where formal access breaks down. Ethnographic and service-design methods can examine waiting rooms, referral letters, digital portals, benefit offices, rehabilitation planning, courts, and employment services as environments with variable affective and epistemic accessibility.

Intervention research could compare standard procedures with psychodynamically accessible redesigns. Examples include low-shame intake letters, stepwise complaint pathways, peer-supported applications, repeated information with relational continuity, trauma-informed legal feedback, shame-sensitive employment coaching, low-penalty re-entry after dropout, and service or prevention messages that acknowledge uncertainty and legitimate mistrust. Outcomes should include not only symptom change but agency: does the person return, ask questions, file the form, negotiate, attend, repair rupture, claim a right, or remain engaged in rehabilitation? Implementation outcomes such as acceptability, feasibility, fidelity, penetration, and sustainability should be included because an accessibility model is clinically meaningful only if services can actually implement it (53).

**Table 5** proposes a staged validation roadmap. The roadmap is designed to prevent premature institutional translation. A concept that is meant to protect dignity can become harmful if applied too quickly as a label. Validation should therefore move from mapping to measurement, from measurement to pilot redesign, from pilot redesign to implementation, and from implementation to prevention outcomes. Complex-intervention and implementation frameworks can help specify intervention components, context, mechanisms, and sustainability before larger trials are justified (53, 55, 56). Trauma-informed implementation principles are also relevant because the model targets procedures that may reproduce threat, humiliation, or loss of control (54).

**Table 5:** Staged validation roadmap and prevention-oriented outcomes.

Stage	Purpose	Possible design	Candidate outcome
Stage 1: Qualitative mapping	Identify where formal access breaks down in lived service trajectories.	Interviews, participatory workshops, ethnography of intake, discharge, complaints, and benefit interfaces.	Map of affective, relational, epistemic, procedural, and temporal barriers; candidate redesign points.
Stage 2: Measurement development	Operationalize conversion factors and accessibility dimensions.	ETMCQ, OPD-3, shame/self-criticism scales, administrative-burden indicators, engagement metrics.	Feasible measurement battery; construct validity; acceptability for service users and staff.
Stage 3: Pilot redesign	Test low-risk modifications of high-friction access points.	Low-shame letters, named contact person, peer-supported forms, teach-back, flexible re-entry after missed appointments.	Improved attendance, comprehension, satisfaction, rights use, and return after rupture.
Stage 4: Implementation study	Assess service-level feasibility and sustainability.	Hybrid implementation-effectiveness design; staff training; fidelity checks; peer co-production.	Acceptability, feasibility, fidelity, penetration, sustainability, and no increase in paternalistic practice.
Stage 5: Prevention outcomes	Evaluate contribution to secondary and tertiary prevention.	Early psychosis, youth hubs, supported employment, rehabilitation transitions, discharge/re-entry pathways.	Reduced delay, dropout, relapse-related disengagement, administrative failure, and service-induced shame or mistrust.

## 10 Ethical safeguards and anti-stigma framework

The greatest risk of the model is misuse. A psychodynamic account of precarity, institutional disengagement, or rights-claiming could be misread as blaming the person, pathologizing poverty, or justifying paternalism. This article rejects those uses.

The model does not diagnose social classes or groups. Terms such as structural vulnerability, state-dependent agency, psychodynamic conversion factors, and psychodynamic accessibility describe interactions between persons and environments. A person may function with high integration in one context and become fragile in another. A service may produce collapse through humiliation, speed, opacity, or coercion. The unit of analysis is the person-in-context.

The model does not replace autonomy with expert control. It argues the opposite: autonomy becomes more real when it is supported under conditions of overload. Supported decision-making, procedural justice, and rights-based mental health care already move in this direction (4–7, 34, 35). Psychodynamic accessibility adds that support must also address affect, shame, trust, temporality, and relational continuity.

The model must remain intersectional and power-sensitive. Affective and epistemic barriers are distributed through class, gender, race, disability, migration status, sexuality, age, geography, and institutional history. Epistemic injustice reminds us that some speakers are granted less credibility and fewer interpretive resources for making their suffering intelligible (43). A psychodynamic model that ignores power would reproduce the very barriers it seeks to reduce.

Inclusive language is part of the intervention. Instead of speaking about fragile people as a fixed category, the manuscript speaks about people in structurally vulnerable states, state-dependent agency, and capacity-constraining contexts. Instead of saying that people choose bad work or bad politics, it asks how choices are constrained by material pressure, internalized shame, relational histories, and inaccessible institutions.

## 11 Advantages over existing approaches

The first advantage is conceptual integration without reduction. Social psychiatry is strong in identifying poverty, exclusion, housing, employment, stigma, and discrimination. Psychodynamic accessibility asks how these factors become embodied and enacted in help-seeking, self-advocacy, trust, and occupational choices without reducing structural inequality to psychology.

The second advantage is precision within the capability approach. The capability approach explains why resources are not enough. Psychodynamic accessibility specifies a subgroup of conversion factors that can be operationalized: shame, self-criticism, defense, affect tolerance, personality functioning, epistemic trust, and dependence on external organizers.

The third advantage is service-design relevance. Existing rights-, recovery-, employment-, and early-intervention models already contain relational and access assumptions, but they do not always name the psychodynamic conversion layer explicitly. This model describes that missing layer and translates it into modifiable design dimensions: affective, relational, epistemic, procedural, and temporal accessibility.

The fourth advantage is explanation of high-functioning vulnerability. Some people function precisely because collapse is too dangerous. They may work, care, publish, commute, or perform reliably while lacking internal permission to rest, complain, or ask for help. A purely symptom-based or administrative model may miss this because it equates visible function with health. Psychodynamic accessibility asks whether function is free, coerced, scaffolded, or self-punitive.

The fifth advantage is a prevention-compatible language. Secondary and tertiary prevention often fail at the point of engagement: a person does not attend, does not return, does not trust, does not complete the form, does not ask the question, or cannot tolerate the shame of needing help. Psychodynamic accessibility makes these points visible as service-design problems rather than as individual non-compliance alone.

## 12 Limitations

The article proposes a theory, not a validated causal model. The hypotheses require empirical testing in diverse cultural, occupational, clinical, legal, and service contexts. The model may be useful only in some settings and may require substantial revision after qualitative and quantitative work.

Psychodynamic terminology can be misunderstood. Words such as defense, structural integration, superego, or personality functioning may sound individualizing or stigmatizing when removed from their clinical context. The manuscript therefore uses contemporary language - state-dependent agency, structural vulnerability, psychodynamic accessibility, and conversion factors - while retaining links to OPD, mentalization, epistemic trust, and process-oriented psychodynamic theory.

The model may be culturally specific. Self-advocacy, shame, autonomy, institutional trust, procedural fairness, and help-seeking are culturally mediated. What counts as accessible in one context may be intrusive, disrespectful, or insufficient in another. Cross-cultural and participatory validation is therefore necessary before implementation.

The model could be misused by institutions to identify people as fragile rather than changing inaccessible systems. Any implementation must therefore prioritize rights, co-production, lived-experience involvement, peer support, and measurable institutional redesign. The primary intervention target is the person-environment interface, not the classification of vulnerable users.

The model also remains incomplete. Labor law, racism, gendered violence, education policy, housing markets, digital media architectures, institutional history, and service capacity remain indispensable explanatory levels. Psychodynamic accessibility is a complementary mechanism layer, not a total theory.

## 13 Discussion and conclusion

Social psychiatry and psychiatric rehabilitation need a precise language for the gap between formal access and supported effective agency. This article proposes psychodynamic accessibility as that language. The model argues that institutions are not truly inclusive if they require stable self-worth, high verbal abstraction, future planning, trust, shame tolerance, and self-advocacy under arousal as hidden entry conditions.

The scientific contribution is the specification of a testable conversion layer. Psychodynamic conversion factors - shame, punitive self-evaluation, defensive functioning under threat, impaired affect tolerance, personality-functioning vulnerabilities, epistemic mistrust or credulity, and dependence on external organizers - may influence whether formal rights, services, employment opportunities, information, and participation formats become usable. These factors do not make people less autonomous. They show why autonomy must be supported in context.

The service-design contribution is equally important. Inclusion should not stop at physical, sensory, digital, or cognitive accessibility. It should also include affective, relational, epistemic, procedural, and temporal accessibility. A rights-based and recovery-oriented system should not ask people to become psychologically ideal users before they are allowed to participate. It should be designed so that people remain able to participate when shame, fear, mistrust, fatigue, and structural vulnerability are already present.

The prevention-oriented implication is cautious but promising. Early-intervention and integrated youth-service models in Australia and Canada show that secondary prevention can be organized around low-threshold, youth-friendly, community-based, and multichannel access (45–49). Psychodynamic accessibility adds a testable question to this agenda: which features of access reduce shame, preserve learnability, stabilize self-advocacy, and make re-entry possible after rupture or dropout? If future studies support this question, psychodynamic accessibility could contribute to secondary and tertiary prevention by redesigning the transitions at which services most often lose the people they are meant to help.

The central claim is therefore modest and practical. Formal access is necessary, but it is not enough. Supported effective agency requires environments that remain usable when human beings are already under load.

## Data Availability

The original contributions presented in the study are included in the article/supplementary material. Further inquiries can be directed to the corresponding author.
